# The portuguese version of the big three perfectionism scale – further validation with adults from the general population

**DOI:** 10.1192/j.eurpsy.2021.1188

**Published:** 2021-08-13

**Authors:** J. Oliveira, A.T. Pereira, A. Araujo, C. Cabaços, J. Azevedo, F. Carvalho, A. Macedo

**Affiliations:** 1 Faculty Of Medicine, University of Coimbra, Coimbra, Portugal; 2 Institute Of Psychological Medicine, Faculty Of Medicine, University of Coimbra, coimbra, Portugal; 3 Institute Of Psychological Medicine, Faculty Of Medicine, University of Coimbra, Coimbra, Portugal

**Keywords:** adults, BTPS, Perfectionism, confirmatory factor analysis

## Abstract

**Introduction:**

Both original Big Three Perfectionism Scale (BTPS; Smith et al. 2016), and the Portuguese version validated with a sample of university students (Lino et al. 2018) evaluates three second-order factors (rigid, self-oriented and narcissistic perfectionism) and ten facets.

**Objectives:**

To confirm the BTPS three-factors-ten-dimensions’ structure in a sample of Portuguese adults from the general population.

**Methods:**

A sample of 467 adults (70.7% females; Mean age=38.44±12.27; range: 25-82) answered the BTPS Portuguese version and other validated perfectionism measures (Multidimensional Perfectionism Scales from Frost and Hewitt & Flett; Self-Presentation Perfectionism Scale). To study the temporal stability a sub-sample of 132 participants completed the BTPS again after approximately five weeks. SPSS and AMOS software was used.

**Results:**

The second order model presented an acceptable fit (X²/df=3.115; TLI=.811; CFI=.825; RMSEA=.067). There was also evidence of a general factor comprising all the 45 items (X²/df=3.127; TLI=.809; CFI=.823; [JA1] RMSEA=.068). The Cronbach alphas of the three factors ranged from a=.88 to a=.92; and facets had a>.70 showing a total of a=.94. Total and dimensional scores showed significant positive and moderate to high correlations with the other perfectionism measures and their test-retest correlation coefficients were r=.85 (p<0.001).

**Conclusions:**

This study confirms the validity and reliability of the Portuguese BTPS underlying three-factors structure. Additionally, we found, for the first time, that BTPS can also be validly and reliably used to measure a global perfectionism construct. It is our intention to develop a shorter version the Portuguese BTPS in the near future.

